# Unlocking the Transcriptional Reprogramming Repertoire between Variety-Dependent Responses of Grapevine Berries to Infection by *Aspergillus carbonarius*

**DOI:** 10.3390/plants13152043

**Published:** 2024-07-25

**Authors:** Charikleia K. Kavroumatzi, Anastasia Boutsika, Paula Ortega, Antonios Zambounis, Dimitrios I. Tsitsigiannis

**Affiliations:** 1 Laboratory of Plant Pathology, Department of Crop Science, Agricultural University of Athens, 11855 Athens, Greece; clio_kv@yahoo.com (C.K.K.); paula.ortega@upc.edu (P.O.); 2 Hellenic Agricultural Organization—DIMITRA (ELGO—DIMITRA), Institute of Plant Breeding and Genetic Resources, 57001 Thessaloniki, Greece; bouanastasia@outlook.com (A.B.); azampounis@elgo.gr (A.Z.); 3 Department of Agro-Food Engineering and Biotechnology, Universitat Politècnica de Catalunya, 08860 Castelldefels, Spain

**Keywords:** *Aspergillus carbonarius*, OTA biosynthesis, immunity, plant–microbe interactions, susceptibility, DEGs, *Vitis vinifera*

## Abstract

*Aspergillus carbonarius* causes severe decays on berries in vineyards and is among the main fungal species responsible for grape contamination by ochratoxin A (OTA), which is the foremost mycotoxin produced by this fungus. The main goal of this study was to investigate at the transcriptome level the comparative profiles between two table grape varieties (Victoria and Fraoula, the white and red variety, respectively) after their inoculation with a virulent OTA-producing *A. carbonarius* strain. The two varieties revealed quite different transcriptomic signatures and the expression profiles of the differential expressed genes (DEGs) highlighted distinct and variety-specific responses during the infection period. The significant enrichment of pathways related to the modulation of transcriptional dynamics towards the activation of defence responses, the triggering of the metabolic shunt for the biosynthesis of secondary metabolites, mainly phenylpropanoids, and the upregulation of DEGs encoding phytoalexins, transcription factors, and genes involved in plant–pathogen interaction and immune signaling transduction was revealed in an early time point in Fraoula, whereas, in Victoria, any transcriptional reprogramming was observed after a delay. However, both varieties, to some extent, also showed common expression dynamics for specific DEG families, such as those encoding for laccases and stilbene synthases. Jasmonate (JA) may play a critical modulator role in the defence machinery as various JA-biosynthetic DEGs were upregulated. Along with the broader modulation of the transcriptome that was observed in white grape, expression profiles of specific *A. carbonarius* genes related to pathogenesis, fungal sporulation, and conidiation highlight the higher susceptibility of Victoria. Furthermore, the *A. carbonarius* transcriptional patterns directly associated with the regulation of the pathogen OTA-biosynthesis gene cluster were more highly induced in Victoria than in Fraoula. The latter was less contaminated by OTA and showed substantially lower sporulation. These findings contribute to uncovering the interplay beyond this plant–microbe interaction.

## 1. Introduction

Grapevine (*Vitis vinifera* L.) is one of the main cultivated temperate crops globally [1], with an annual yield of its fruits around 74.9 million tons for 2022 [2]. However, a significant amount of the yield is lost annually and various diseases represent important problems for viticulture worldwide [3]. 

Among the various fungal pathogens that caused severe damages on grapevine, the filamentous fungus *Aspergillus carbonarius* (Bainier), Thom (1916) that belongs to the black *Aspergillus* section *Nigri* [4] is reported to cause extensive grape berry decay in vineyards [5,6,7,8]. Thus, in Europe, South America, Australia, and the USA, grapes, wine, and raisins are widely infected by this necrotrophic fungus [9,10,11,12]. Apart from the potential berry rotting, this fungus produces mycotoxins in grapes and wine, including ochratoxin A (OTA) [7,13,14,15,16,17,18,19,20]. The European Commission set a maximum detection limit of 2 μg/kg for OTA contamination, one of the most dangerous mycotoxins [21,22]. Various parameters, such as the vineyard location, pest infestation [23], soil type, weather conditions, and susceptibility of grape varieties, as well as the OTA production, may influence the disease severity during fungal colonization [24].

There is currently little knowledge on the mechanisms driving *A. carbonarius* infection in grape berry bunching. Nonetheless, it has been suggested that the physical and chemical properties of berries play a major role in defining variations in pathogen incidence and OTA production amongst grape varieties [7,25]. Furthermore, it has been suggested that phenolic chemicals, acidity, and total sugars may influence the prevalence of filamentous fungi [26]. In response to *A. carbonarius* infection, grapes seem to activate several variety-specific responses recruiting an array of defence mechanisms through the triggering of multidimensional biochemical processes [7] putatively mediated by effector- and pathogen-associated molecular-pattern-triggered immunity (ETI and PTI, respectively) at the plant–pathogen interface [27,28]. Commonly, the downstream cell signaling cascade involves overlapping metabolic hubs that determine the resistance or susceptibility to pathogen challenges [29]. In this aspect, hormonal balancing is crucial for triggering the transcriptional co-ordination of several genes, and, particularly, the induction of pathogenesis-related (PR) proteins along with a chemically diverse class of specialized defence metabolites called phytoalexins, which are crucial components towards the activation of immunity responses [30,31].

Currently, in order to obtain valuable insights into the interactions between plant hosts and pathogens, RNA-sequencing (RNA-seq) technology has been widely used [32]. This technology allows for the accurate capture of the complex host transcriptome responses during pathogen infection, elucidating plant defence mechanisms and the regulatory networks governing these processes. As a result, hundreds of transcriptome profiling studies have been carried out to date, proving that RNA-seq is an advanced method for figuring out the regulation of molecular mechanisms underlying these interactions [32,33]. However, the transcriptional dynamics of grape berries after inoculation with *A. carbonarius* remain widely unknown.

In the present study, the comparative transcriptome profiles between two grape varieties (a white and a red one) were revealed after their inoculation with a virulent OTA-producing *A. carbonarius* strain during a three-day inoculation period. Susceptibility in terms of conidia quantification and OTA accumulation were also investigated in the end of a seven-day inoculation period with the pathogen. Furthermore, the *A. carbonarius*-induced genes were also tracked in order to elucidate the fungal genes involved in sporulation, pathogenicity, and, mainly, OTA production. Comprehending the mechanisms underlying variety-specific responses upon infection with this phytopathogen might improve grape bunch rot management.

## 2. Results

### 2.1. Fungal and Ochratoxin Quantification

**Figure 1:**
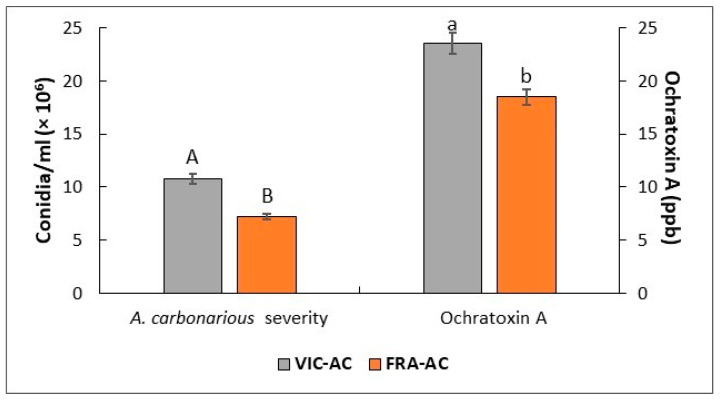
Mean severity (conidia/mL) of *A. carbonarius* and ochratoxin A content (ppb) in inoculated grape berries of white variety Victoria (VIC-AC treatment) and red variety Fraoula (FRA-AC treatment) upon a 7-day inoculation period. Bar plots represent mean values of three biological replicates ± standard errors per treatment considered in the study. Different letters reported on each bar indicate statistical differences among treatments.

*A. carbonarius* was grown in inoculated berries of both varieties upon the seven-day inoculation period. All developed colonies were identical to *A. carbonarius* based on their color, morphology, and conidia characteristics, whereas any symptoms were absent in the control, mock-inoculated berries. The inoculation treatments between the two varieties significantly impacted the fungal growth, sporulation, and OTA biosynthesis (Figure 1). Therefore, moldy berries of the Victoria variety (VIC-AC treatment) produced a more significant number of fungal spores/mL (*p* ≤ 0.05), expressed as conidia /mL (×10^6^), compared to those of the Fraoula variety. The higher OTA content was also detected in the Victoria variety, ranging approximately up to 23.52 ± 2.02 ppb (μg/L) with significant differences comparable to the Fraoula variety according to Tukey’s multiple test (*p* < 0.05).

### 2.2. Transcriptomics and Differentially Expressed Genes on Grape Berries

**Figure 2:**
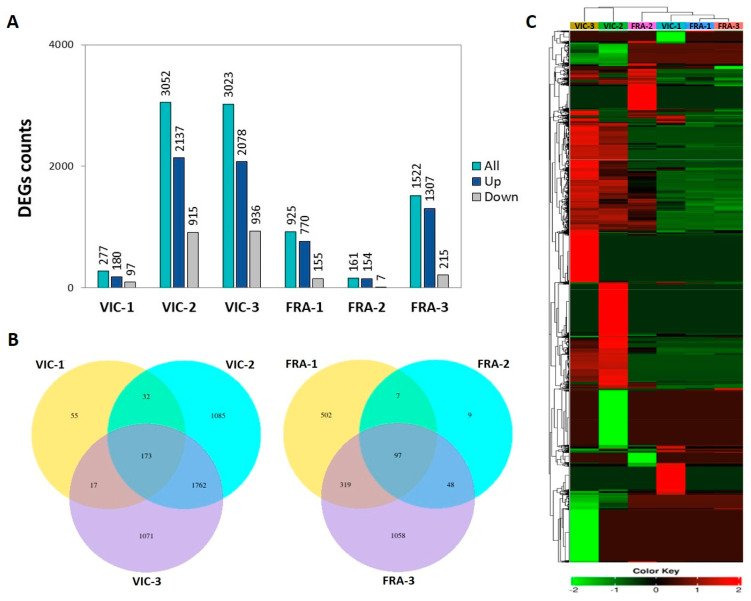
(**A**) Gene number of up-/downregulated DEGs among the six different comparison groups (VIC-1, VIC-2, VIC-3, FRA-1, FRA-2, and FRA-3) at 1, 2, and 3 dai. (**B**) Venn diagrams showing DEGs commonly regulated across the three comparison groups for each variety. DEGs were identified by comparing inoculated and controls over each variety/time point. (**C**) Heatmap of the hierarchical clustering of grape DEGs among the six comparison groups (VIC-1, VIC-2, VIC-3, FRA-1, FRA-2, and FRA-3) using the Euclidean distance based on median normalized log2foldchanges values.

To decipher the mechanism of the transcriptomic profiles in the two grape varieties after an *A. carbonarius* challenge, a dual RNA-seq analysis was conducted upon 36 berry samples. Thus, samples consisted of berries inoculated with the pathogen (VIC-AC and FRA-AC treatments) or from untreated, mock-inoculated berries (VIC-CT and FRA-CT treatments), employing three biological replicates for each treatment across three time points upon inoculation (1, 2, and 3 dai). Approximately 86% of the 1.242.824.810 high-quality paired-end reads were uniquely mapped to the grape genome assembly (Table S1). Pairwise comparisons of expression profiles between inoculated and mock-inoculated berries (controls) within each variety and each time point were performed. Thus, six comparison groups, namely, VIC-1, VIC-2, and VIC-3 for the Victoria variety and FRA-1, FRA-2, and FRA-3 for the Fraoula variety, respectively, for the 1, 2, and 3 dai, were assigned. DEGs within each comparison group were determined using a threshold of log2foldchange ≥ 1, and an adjusted *p*-value ≤ 0.05 (Table S2). The number of up-/downregulated DEGs in each of the six comparison groups was counted and shown in Figure 2A. Furthermore, the correlation between the amount of up- and downregulated DEGs in the six comparison groups is visually represented by volcano plots (Figure S1). The highest number of DEGs was observed in the VIC-2 comparison group (2137 up- and 915 downregulated). At the earliest time point in the VIC-1 comparison group, only 277 DEGs were induced, whilst, in the FRA-2 group, 161 DEGs were induced with seven of them to be suppressed. In all six comparison groups, the number of upregulated DEGs was higher compared to the downregulated DEGs. For the Victoria variety, the highest number of upregulated DEGs was recorded in the VIC-2 group, whilst, for the Fraoula variety, it was in the FRA-3 group. Venn diagrams were also generated showing the overlapping number of DEGs between the three comparison groups per variety (Figure 2B). The results showed that only 173 and 97 DEGs among the three comparison groups for each cultivar, Victoria and Fraoula, respectively, sustained a common co-expression across all time points. Furthermore, the highest number of specifically induced DEGs among the three time points for each cultivar was recorded in the VIC-2 and FRA-3 comparison groups. A heatmap showing the hierarchical clustering of grape DEGs across the six comparison groups (Figure 2C) suggests a dynamic and time-dependent transcriptional reprogramming of grape berries upon infection based on the expression patterns of the RNA-seq data. 

### 2.3. Gene Ontology Categorization and KEGG Enrichment Analysis of DEGs

Based on the GO term enrichment analysis, DEGs were assigned to significant functional annotations (Figure S2; Table S2). Among the comparison groups of both varieties, the term “copper ion binding” was found to be highly enriched and constitutively upregulated. Over the infection period, the number of DEGs assigned to this term was progressively increased. However, it is worth mentioning that a significant shift towards the GO terms’ enrichment was recorded only in the VIC-2 and VIC-3 comparison groups. In these groups, the GO term “defense response” was constitutively enriched and mainly upregulated in the Victoria variety. On the contrary, in the Fraoula variety, a diminishing of significant GO term enrichment was observed in the FRA-2 group. On the other hand, the term “defense response” and other biological processes were significantly enriched in the FRA-1 and FRA-3 groups. Notably, in the FRA-1 group, GO terms that correspond to molecular functions, such as those of “pectinesterase activity” and “xyloglucan:xyloglucosyl transferase activity” were significantly enriched with the majority of related DEGs to be upregulated. A similar enrichment pattern was also revealed in the VIC-2 and FRA-3 group regarding the GO terms “DNA binding transcription factor activity” and “transcription regulator activity”, which were constitutively enriched and mainly upregulated across these two groups (Figure S2; Table S2).

**Figure 3:**
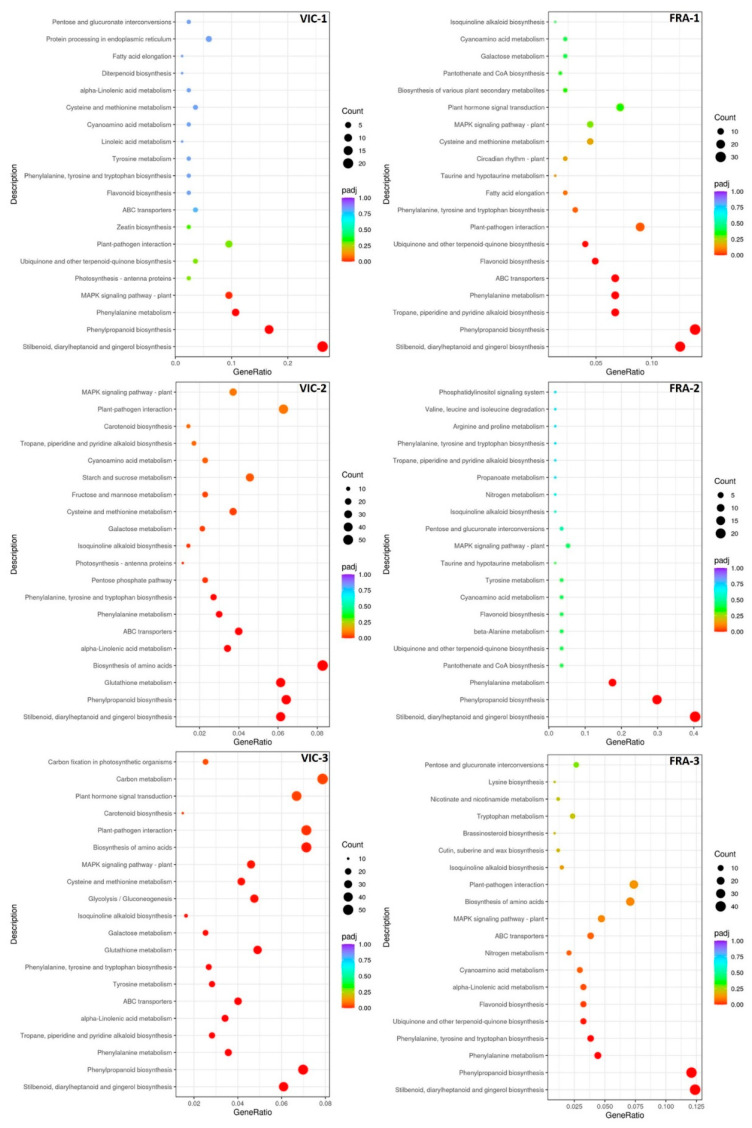
Enrichment scatter plots based on the classification of the grape berries’ DEGs in KEGG pathways across the six comparison groups (VIC-1, VIC-2, VIC-3, FRA-1, FRA-2, and FRA-3). The counts of the DEGs being annotated in the corresponding KEGG pathways are shown, whereas the significant size (adjusted *p*-value, padj) of the enrichment is indicated by a color.

In the Victoria variety, a significant KEGG enrichment was mainly observed in the VIC-2 and VIC-3 groups, whereas, in the Fraoula variety, it was observed in the FRA-1 and FRA-3 groups (Figure 3; Table S2). However, across all six comparison groups, the KEGG pathway of “stilbenoid, diarylheptanoid, and gingerol biosynthesis” was constitutively enriched and highly activated at all three times points for both varieties. Furthermore, the “phenylpropanoid biosynthesis”, and “phenylalanine metabolism” pathways were highly enriched in the six comparison groups. Pathways related to “MAPK signaling pathway”, “plant–pathogen interaction”, “alpha-linolenic acid metabolism”, and “biosynthesis of amino acids” were constitutively enriched in the VIC-2 and VIC-3 groups. In addition, significant enriched pathways, such as “plant–pathogen interaction”, “ubiquinone and other terpenoid-quinone biosynthesis”, and “flavonoid biosynthesis”, were constitutively enriched in the FRA-1 and FRA-3 groups (Figure 3; Table S2).

### 2.4. Transcriptional Changes on Infected Grape Berries by A. carbonarius

**Figure 4:**
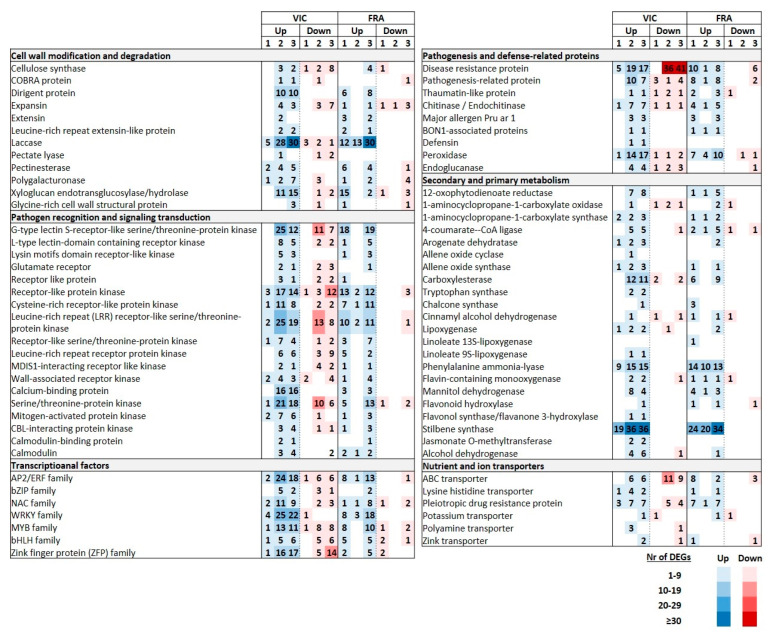
Heat map of selected upregulated (Up) and downregulated (Down) key DEGs across the six comparison groups (VIC-1, VIC-2, VIC-3, FRA-1, FRA-2, and FRA-3). The numbers of the differentially expressed transcripts are reported for both downregulated (red color scale) and upregulated (blue color scale) gene groups at 1, 2, and 3 dai that correspond to VIC-1 and FRA-1, VIC-2 and FRA-2, and VIC-3 and FRA-3 comparison groups, respectively.

In the VIC-2, VIC-3, FRA-1, and FRA-3 comparison groups, DEGs related to cell-wall processes were mainly upregulated, while an almost absence of induction was reported in the VIC-1 and FRA-2 groups (Figure 4; Table S3). Thus, DEGs encoding dirigent protein (DIR) and leucine-rich repeat containing extensin (LRX) were constitutively upregulated in the 2 and 3 dai for the Victoria variety, and in the 1 and 3 dai for Fraoula variety. This differential trend was also observed in the case of the upregulation profile of xyloglucan endotransglucosylase/hydrolase (XTH) and pectinesterase (PME) encoding DEGs. DEGs encoding cellulose synthase (CesA) were mainly downregulated at the latest time point in the Victoria variety (VIC-3 group), and constitutively upregulated in 3 dai in the Fraoula variety (FRA-3 group). Finally, it was noteworthy that DEGs encoding laccase (LAC) were highly upregulated in all six comparison groups.

A similar expression profile was evident among the two varieties and their comparisons groups regarding DEGs encoding a plethora of pathogen recognition receptors (PRRs) associated with pathogen perception and signaling transduction (Figure 4; Table S4). Most of these genes, such as those encoding members of *G*-type lectin receptor kinases (GsSRKs) and the LRR receptor-like serine/threonine-protein kinase family (LRR-RLKs), cysteine-rich receptor-like protein kinases (CRKs), and serine/threonine-protein kinases (STPKs), were mainly upregulated in the VIC-2, VIC-3, FRA-1, and FRA-3 comparison groups. However, in the FRA-1 and FRA-3 groups, the expression patterns of receptor-like kinase (RLK)- and receptor-like protein (RLP)-encoding genes showed a higher modulation towards upregulation in comparison to VIC-2 and VIC-3 groups where a number of those DEGs were suppressed upon *A. carbonarious* infection. Thus, except for DEGs encoding calcium-binding proteins (CBPs), RLKs containing lysin motifs (LysM RLKs), and CBL-interacting serine/threonine-protein kinases (CIPKs), which were constitutively upregulated in the VIC-2, VIC-3, FRA-1, and FRA-3 groups, DEGs encoding wall-associated receptor kinases (WAKs) were constitutively upregulated only in the FRA-1 and FRA-3 groups.

An abundant number of DEGs encoding pathogenesis- and defense-related proteins were highly induced in the VIC-2, VIC-3, FRA-1, and FRA-3 comparison groups (Figure 4; Table S5). Interestingly, a considerably high number of DEGs related to disease resistance proteins were downregulated in the VIC-2 and VIC-3, whereas those genes were mainly upregulated in the FRA-1 group. Particularly, peroxidase and chitinase/endochitinase encoding DEGs were mainly upregulated in the VIC-2, VIC-3, FRA-1, and FRA-3 groups. In these comparison groups, DEGs encoding Pru ar (major allergen Pru ar 1) and BON1-associated proteins (BAPs) were constitutively upregulated. Finally, defensin encoding a small cysteine-rich cationic protein was only upregulated in the Victoria variety at 2 and 3 dai (the VIC-2 and VIC-3 groups).

The expression profiles among transcription factor (TF) hubs revealed a high upregulation of members of the AP2/ERF, NAC, MYB, ZFP and WRKY families, particularly across the VIC-2, VIC-3, FRA-1, and FRA-3 groups (Figure 4; Table S6) However, a considerable number of those TF-encoding members of the AP2/ERF, NAC, MYB, and ZFP families were also suppressed on these groups, especially in the VIC-2 and VIC-3 groups. It is worth mentioning that all WRKY-encoding DEGs were upregulated in the VIC-2, VIC-3, FRA-1, and FRA-3 groups, and mostly in the VIC-2 and VIC-3 groups.

Several DEGs associated with the triggering of primary and secondary metabolism were mostly induced in the VIC-2, VIC-3, FRA-1, and FRA-3 groups (Figure 4; Table S7). Among them, DEGs encoding 12-oxophytodienoate reductase (OPR), 1-aminocyclopropane-1-carboxylate synthase (ACS), 4-coumarate–CoA ligase (4CL), allene oxide synthase (AOS), carboxylesterase (CXE), and mannitol dehydrogenase (MTD) were mainly upregulated at these four comparison groups. It is worth mentioning the constitutive upregulation of a high number of DEGs encoding stilbene synthase (STS) and phenylalanine ammonia-lyase (PAL) across all six comparison groups upon *A. carbonarious* inoculation. Different types of linoleate lipoxygenase (LOX)-encoding DEGs were upregulated in the VIC-2, VIC-3, and FRA-1 groups. DEGs encoding jasmonate O-methyltransferase (JMT), arogenate dehydratase (ADT), and tryptophan synthase (TRPS), as well flavanone 3-hydroxylase (F3H) were mainly upregulated in the VIC-2 and VIC-3 groups, while DEGs encoding chalcone synthase (CHS) were upregulated mainly in the FRA-1 group.

Nutrient and ion transporters encoding DEGs were mainly induced in the VIC-2, VIC-3, FRA-1, and FRA-3 groups, such as members of the ABC transporters and lysine histidine transporter (LHT) gene family (Figure 4; Table S8). Members of the pleiotropic drug resistance gene family was induced across all six comparison groups, mostly by an upregulation expression pattern. 

### 2.5. A. carbonarius Expressed Genes during Grape Infection 

**Figure 5:**
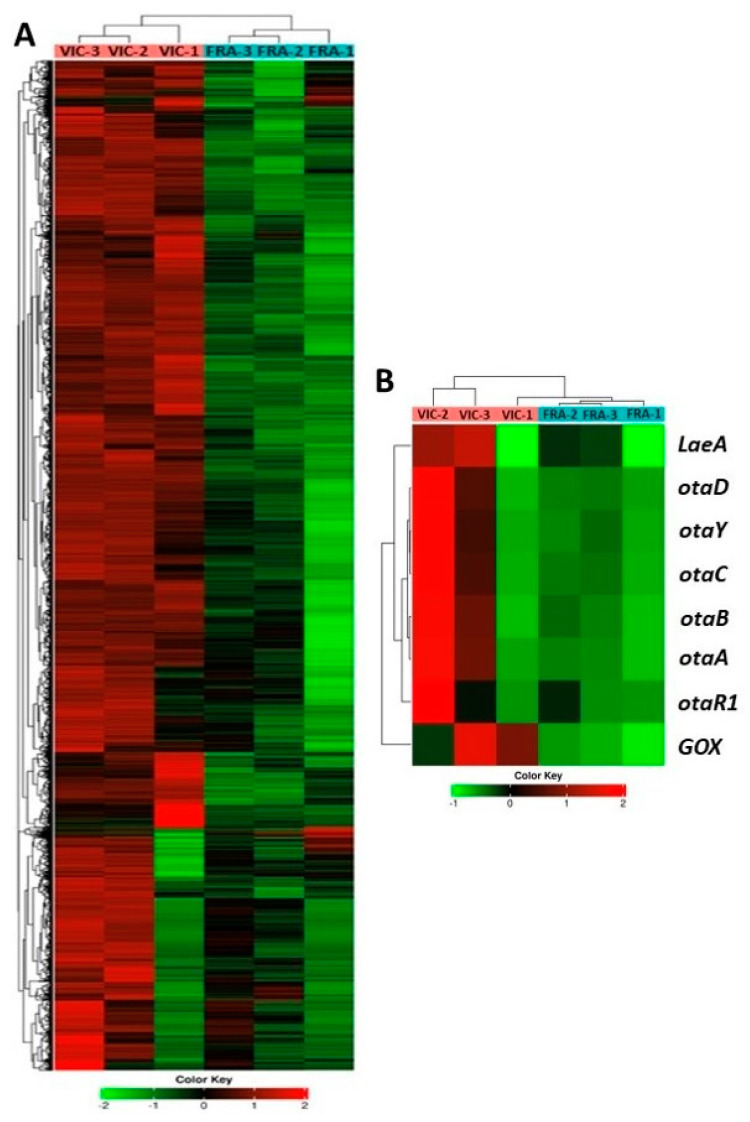
(**A**) Heatmap of the hierarchical clustering of *A. carbonarius*-expressed genes based on their average counts of normalized reads across the six comparison groups (VIC-1, VIC-2, VIC-3, FRA-1, FRA-2, and FRA-3). (**B**) Sub-heatmap showing the expression patterns of the hub genes directly associated with regulation of OTA-biosynthesis gene cluster across the six comparison groups.

Notably, a high differential profile of *A. carbonarius*-expressed genes was recorded between the two varieties. A heatmap of the hierarchical clustering of pathogen-expressed genes based on the average counts of normalized reads across the six comparison groups is shown in Figure 5A. A number of genes associated with pathogen growth, sporulation, pathogenicity, and OTA accumulation were mainly induced in the Victoria variety, particularly in the VIC-2 and VIC-3 groups, peaking at the latest time point (Table S9). On the contrary, in the Fraoula variety, a near absence of expressed genes was recorded in the FRA-1 and FRA-2 groups with only a slight increase in FRA-3, which, however, was lees abundant in comparison with the expression profiles in the Victoria variety over the three time points. Thus, genes associated with *A. carbonarius* growth and pathogenicity were mainly detected in the Victoria variety (VIC-1, VIC-2, and VIC-3 groups). For example, four members of the *stuA* genes encoding a cell-pattern-formation-associated protein involved in conidiation and sporulation, along with an *MYB TF* encoding a conidiophore development protein (FlbD), were highly detected mostly in the Victoria variety. Additionally, genes serving as structural components of the ribosome, as well as genes encoding ribosome biogenesis proteins, such as Erb1 (A0A1R3S351), Urb1 (A0A1R3REW6), and Ytm1 (A0A1R3RTS3), were detected to a lesser extent in Fraoula compared to the Victoria variety across the three time points. A number of genes encoding hydrolytic or carbohydrate-active enzymes (CAZymes), which are related to plant cell-wall disruption, were also identified in a higher extent in the three comparison groups of the Victoria variety. Particularly, genes encoding endo-polygalacturonases were highly detected in the Victoria variety and were less expressed in Fraoula. Similar expression patterns were revealed in terms of several lytic genes, such as those encoding peroxidase involved in lignin degradation and pectinesterase involved in pectin degradation, and a gene encoding the Ecp2 effector (A0A1R3R6H2).

Particularly, in terms of the transcriptional patterns directly associated with the regulation of the pathogen OTA-biosynthesis gene cluster, the expression profiles of *OTApks* (*A0A1R3RGK0*), *OTAnrps* (*A0A1R3RGK1*), *OTAp450* (*A0A1R3RGJ7*), *OTAhal* (*A0A1R3RGJ2*), and *OTAbZIP* (*A0A1R3RGK4*) encoding for a polyketide synthase (OtaA), a nonribosomal peptide synthetase (OtaB), a cytochrome P450 monooxygenase (OtaC), a flavin-dependent halogenase (OtaD), and a member of bZIP TFs (OtaR1), respectively, were revealed to have a high co-expression in the VIC-2 comparison group (Figure 5B; Table S9). A less detectable expression profile for these six genes representing the OTA cluster was revealed in the VIC-3 group, whereas, notably, an even lower expression of these genes was recorded in the FRA-2 and FRA-3 groups. Additionally, the *otaY* gene (A0A1R3RGJ9) encoding a cyclase that was putatively involved in the OTA gene cluster was mainly detected in the VIC-2 and VIC-3 groups. Similarly, the velvet complex subunit *laeA* (A0A1R3RLQ3) that belongs to LaeA methyltranderase family, which is involved in both sporulation and the regulation of the production of secondary metabolites, was mainly detected in the VIC-2 and VIC-3 groups. A gene encoding a glucose oxidase (GOX) belonging to the GMC oxidoreductase family (A0A1R3RM75), which is controlling the gluconic acid (GLA) accumulation and acidization of the host tissue during fungal growth and is required for *A. carbonarius* pathogenicity, was significantly expressed in both varieties with a higher extent in the VIC-1, VIC-2, and VIC-3 groups compared to FRA-1, FRA-2, and FRA-3. Notably, this gene was one of the most highly expressed *A. carbonarius* genes across all six comparison groups. 

### 2.6. Validation of DEGs by RT-qPCR

Six DEGs were randomly chosen for the validation of the RNA-seq data through a real-time quantitative PCR (RT-qPCR) approach. All the gene-specific primers can be accessed in Table S10. These RT-qPCR assays were conducted to validate the log2foldchange values of the respective RNA-seq data. As shown in Figure S3, the expression patterns of all the chosen DEGs constitutively reflected those of the RNA-seq data.

## 3. Discussion

This study’s RNA-seq data are consistent with the dynamic metabolomic fingerprints that are observed across red and white grape varieties in response to *A. carbonarius* infection [7]. In our study, the variety-dependent transcriptomic profiles were revealed, which were motivated in a time-dependent mode. Particularly, grape berries of the Fraoula variety were able to induce a level of basal defense at the earliest time point of inoculation (1 dai) compared to the Victoria variety. In contrast, in the latter variety, any defense responses were observed after a delay and not earlier than 2 dai. Notably, after the early transcriptional reprogramming in the Fraoula variety (the FRA-1 group), a diphasic reprogramming occurred with a drop in DEGs induction in the FRA-2 group, and a subsequent increase in the FRA-3 group. Furthermore, the transcriptional changes in the red variety seem to be more compact, showing, to some extent, a less expanded transcriptional reprogramming compared to those in the white variety that showed a broader modulation of the transcriptome. Previously, red varieties showed more generalized biochemical modulation upon *A. carbnarius* inoculation in comparison to the white varieties [7]. Taking into consideration that the Victoria variety revealed a significantly higher degree of fungal growth by means of conidia sporulation, as well as a higher OTA content, we are tempted to speculate that this variety is more susceptible to *A. carbonarius*.

The cell wall constitutes the structural barrier in response to a pathogen attack, contributing to the initiation of a plant defense repertoire [34,35], whereas its disassembly and degradation contributes to its susceptibility to pathogen infection [36,37]. The proportion of upregulated *CesA*s genes that contribute to impeding the pathogen penetration by cellulose synthesis was higher in the FRA-1 and FRA-3 groups than in the VIC-2 and VIC-3 groups. On the other hand, an adequate number of *DIR*, *LRX*, and *XTH* genes were mainly upregulated in the VIC-2, VIC-3, FRA-1, and FRA-3 groups. This indicates a shift towards a partial deployment of defense responses related to cell-wall modification in a time-dependent mode between the two varieties, as these genes are associated with hampering pathogen penetration by the thickening, stiffening, lignification, and fortification of cell walls [34,35,37,38]. Notably, a high repertoire of *LACs* genes was mainly upregulated in the VIC-1, VIC-2, and VIC-3 groups, while they were constitutively upregulated in the FRA-1, FRA-2, and FRA-3 groups. Their induction was attributed to the highly enriched GO term “copper ion binding” in all six comparison groups. In plants, laccases participating in the lignification of cell-wall appositions is a conserved basal defense mechanism [39]. It is also worth mentioning that, in resistant apple genotypes against *Pythium ultimum* infection in roots, the increased activity of laccase appears to exist prior to pathogen exposure [40]. Furthermore, the overexpression of the *LAC* gene enhanced the *Verticillium* wilt resistance in cotton [41]. A similar overexpression of such a gene in cotton increased lignification and enhanced JA biosynthesis [42]. On the other hand, in both varieties, the upregulation of most *PME* genes, which are involved in cell-wall loosening [37], may putatively facilitate the pathogen colonization. Overall, these results indicate that cell-wall modification and degradation processes were activated earlier in the red variety in response to *A. carbonarius* inoculation.

An abundant repertoire of RLK- and RLP-encoding DEGs involved in plant–microbe interaction, pathogen recognition, and signaling were upregulated mainly in the VIC-2, VIC-3, FRA-1, and FRA-3 groups, even though a considerable number of such DEGs were also suppressed in Victoria-related groups. Particularly, the upregulation of an array of *GsSRK*, *LRR-RLK*, *CRK*, *RLK,* and *WAK* genes was observed in the FRA-1 group. Several *RLK* genes were previously shown to be activated in immune responses against necrotrophic fungal pathogens [37,43], such as *GsSRKs* [38,42]. Furthermore, *CRK* genes are involved in the regulation of immune signaling pathways [43]. Previously, it was also reported that *WAK* genes were upregulated during infection with *B. cinerea* in ripe strawberry fruits, lettuce, and rose petals [34,38,44].

Upon *A. carbonarius* infection, a large set of TFs were induced across the inoculation period, such as those belonging to the AP2/ERF, NAC, MYB, ZFP, and WRKY families. Members of these TF families play a pivotal role in the orchestration and regulation of defense mechanisms in plants [37,39,45,46,47,48,49,50]. In our study, the transcriptional reprogramming of TF-encoding DEGs was driven by a different and time-dependent expression profile between the two varieties. Thus, the majority of them were mainly upregulated across the VIC-2, VIC-3, FRA-1, and FRA-3 groups. However, a considerable number was also suppressed in these groups, particularly in the VIC-2 and VIC-3 groups, which further indicates the suppression of defense mechanisms in the Victoria variety. As a result, the GO terms ‘DNA binding transcription factor activity’ and ‘transcription regulator activity’ were significantly enriched and mainly upregulated in the VIC-2 and FRA-3 groups. Members of the bHLH family are classified as JA-mediated transcriptional regulators [51], whereas ERFs integrate signals involving JA and ET to regulate molecular responses upon fungal inoculation [52]. Furthermore, ZFP and WRKY members contribute to the regulation and modulation of defense responses against fungal attacks [46,53,54]. Notably, WRKY-encoding DEGs were constitutively upregulated in the VIC-2, VIC-3, FRA-1, and FRA-3 groups. It is also interesting to note that a considerable number of MYB family members were upregulated in the FRA-1 and FRA-3 groups. These TFs modulate defense networks and promote the induction of flavonoid accumulation towards the early activation of PTI in response to a fungal attack [55].

It is known that the reprogramming of secondary metabolism activates host defense responses and pathways to tackle a fungal challenge [56,57]. Particularly, in our study, this metabolic shunt was mainly revealed no earlier than at 2 dai in the Victoria variety, whereas, in contrast, in the Fraoula variety, a diphasic modulation was revealed with an early activation at 1 dai followed by a later one at 3 dai. *STSs* are involved in the KEGG pathway of “stilbenoid, diarylheptanoid, and gingerol biosynthesis”, which was constitutively enriched and highly activated at all three time points for both varieties. Their encoded enzymes are key components in the biosynthesis and accumulation of stilbene phytoalexins, a small class of plant secondary metabolites that are derived from the phenylpropanoid pathway and induce defense mechanisms in grape [58]. Transgenic plants expressing *STSs* enhanced the disease resistance to pathogens, such as *Erwinia carotovora*, *Pyricularia oryzae*, and *B. cinerea* [59,60,61]. Even though the expression of an *STS* gene enhanced the resistance against powdery mildew in grapevine [62], we did not observe any metabolomic modulation of stilbenes in response to an infection by *A. carbonarius* in grapevine berries [7]. In our study, it is worth mentioning the constitutive co-upregulation of a high number of *STS* genes across all six comparison groups. A similar expression pattern was also observed in terms of the induction of *PAL* genes that encode the key enzyme in the phenol biosynthesis pathway and contributed to the high enrichment of ‘phenylpropanoid biosynthesis’ and ‘phenylalanine metabolism’ KEGG pathways in all six comparison groups. Furthermore, *4-CL* genes that participate in the induction of ‘phenylpropanoid biosynthesis’ and ‘ubiquinone and other terpenoid-quinone biosynthesis’ KEGG pathways were mainly upregulated in the VIC-2, VIC-3, FRA-1, and FRA-3 groups.

Previously, the accumulation of phenylpropanoids and terpenoids, along with phytoalexins, was observed in grape berries of the white and red varieties upon *A. carbonarius* infection [7]. In order to overcome the host defenses, mannitol is secreted by a number of fungal pathogens [63]. This response is necessary for their pathogenicity, while mannitol-deficient mutants of *Alternaria alternata* showed a drop in their pathogenicity on tobacco [64]. In turn, plants activate pathogen-induced MTDs to efficiently catabolize the pathogen’s secreted mannitol [63]. Our RNA-seq data revealed a constitutive upregulation of MTD genes, which may contribute to the activation of defense responses upon an *A. carbonarius* challenge. Similarly, DEGs encoding OPR enzymes were upregulated, whereas their activation is promoting the biosynthesis of various secondary metabolites, JA biosynthesis, and the ‘alpha-linolenic acid metabolism’ KEGG pathway [65]. The latter primary biosynthetic pathway was constitutively enriched in the VIC-2 and VIC-3 groups, also through the upregulation of a few AOSs, LOXs, and linoleate 9S-lipoxygenase-encoding genes.

It is worth mentioning that several of these genes are also involved in JA biosynthesis, which is associated with crucial regulating roles in defense responses against necrotrophic fungi [34,57,66,67]. In turn, this entails that JA might play a crucial role in signaling transduction-mediated responses, at least in the Victoria variety. This hypothesis is further reinforced by the upregulation of *JMT* genes in the VIC-2 and VIC-3 groups. In addition, *CXE* genes were mainly upregulated in the VIC-2, VIC-3, FRA-1, and FRA-3 groups. These genes were shown to improve disease resistance against fungal pathogens [68,69].

On the other hand, flavonoids have been shown to have antifungal properties against fungi and their accumulation is crucial for triggering defense responses in plants [70]. The constitutive upregulation of DEGs encoding *CHS* genes might further contribute to the enrichment of the “flavonoid biosynthesis” KEGG pathway in the FRA-1 and FRA-3 groups. Furthermore, DEGs encoding *ADT* and *TRPS* genes were mainly upregulated in the VIC-2 and VIC-3 groups and may contribute to the enrichment of the “biosynthesis of amino acids” KEGG pathway in the Victoria variety. The induction of such DEGs might be manipulated by *A. carbonarius* for obtaining a further nitrogen source for pathogenesis. On the other hand, by providing additional precursors for the biosynthesis of secondary metabolites during infection, this reprogramming of primary metabolism may activate grape defense responses [37,71].

A few members of a PAMP-induced gene, named respiratory burst oxidase homolog (Rboh), which regulates the accumulation of reactive oxygen species (ROS) and redox homeostasis [37,72], were constitutively upregulated at both varieties across the inoculation period. In addition, another scavenging strategy seems to be transcriptionally mediated by the induction of a plethora of glutathione-S-transferase-encoding genes that directly participate in the ROS scavenging pathway [73], and which were mainly upregulated in the VIC-2 and VIC-3 groups.

In our study, the induction of an abundant number of DEGs encoding defense-related and PR proteins was revealed, particularly in the VIC-2, VIC-3, FRA-1, and FRA-3 groups. The induction of such genes further indicates the delay in immunity responses in the Victoria variety. Furthermore, the expression profiles were quite distinct between the two varieties. Thus, in the VIC-2 and VIC-3 groups, a considerable number of these genes, particular of those encoding disease resistance proteins, were shown to have a pronounced tendency of repression, whereas such genes were upregulated mainly in the FRA-1 group. The upregulation of defensin-encoding genes was observed in the VIC-2 and VIC-3 groups. It is worth mentioning that, in both varieties, DEGs encoding homologs of major allergen Pru ar 1 proteins, belonging to the PR-10 group family, were constitutively upregulated in the VIC-2, VIC-3, FRA-1, and FRA-3 groups. These genes interact with metabolic components of flavonoid biosynthesis and participate in defense signaling regulation enhanced by JA [74]. Previously, such genes were also induced in fruits challenged with *B. cinerea* [75]. On the other hand, DEGs encoding BON1-associated proteins (BAPs) were constitutively upregulated in both varieties. These genes are negative regulators of immune responses [76], which suggests that defense responses were also suppressed during the inoculation period. Furthermore, DEGs encoding members of DMR6-like oxygenases were highly upregulated, mainly in the VIC-2 and VIC-3 groups, indicating that defense responses were further suppressed in the Victoria variety contributing to *A. carbonarius* susceptibility. These DEGs are known negative regulators of immunity acting as sensitivity factors of basal defense responses [77].

DEGs encoding transporters were also induced mainly in the VIC-2, VIC-3, FRA-1, and FRA-3 groups. Several of them, such as those encoding LHT transporters, might have manipulated by *A. carbonarius* for its own needs to obtain nutrients from decayed host cells [75]. Furthermore, DEGs encoding ABC transporters such as those of the G family, which are involved in the transport of secondary metabolites in plants challenged by pathogens [78], were highly suppressed in the VIC-2 and VIC-3 groups.

*A. carbonarius* is the main fungus that causes OTA contamination in grapes, especially in the Mediterranean basin [13,79]. In this study, we discovered an array of highly induced *A. carbonarius* genes that may allow us to clarify the fungal genes implicated in pathogenesis during the inoculation period. Thus, genes associated with *A. carbonarius* growth and pathogenicity were mainly detected in the Victoria-related groups (VIC-1, VIC-2, and VIC-3). Among them, we detected four genes encoding StuA proteins, which are involved in conidiation and sporulation [80], as well as an MYB TF that encodes a FlbD, which is a conidiophore development protein [81]. Ribosome-biogenesis-related genes, such as *erb1, urb1,* and *ytm1*, that control the growth of fungal cells were detected at all three time points, although to a lesser extent in Fraoula than in the Victoria variety. Furthermore, the higher susceptibility of the Victoria variety against *A. carbonarius* is highlighted by the fact that genes linked to pathogenicity pathways, such as those involved in the production of secondary metabolites, plant CWDEs (e.g., pectin lyase, endo-polygalacturonase, and pectineasterase), and biopolymer-degrading CAZymes, were either not detected or were detected with lower read counts in the Fraoula than in the Victoria variety. *A. carbonarius* colonization on grape berries coincided with the high expression of genes encoding virulence factors, including effector proteins such as those of the Ecp2 family, mostly in Victoria-related groups.

To date, the putative OTA-biosynthesis gene cluster in *A. carbonarius* is well-refined, including the genes *otaA*, *otaB*, *otaC*, *otaD*, *otaR1*, and *otaY* [13,14,15,16,17,18,19,20]. All these genes were significantly expressed, particularly in the VIC-2 group and, to a lesser extent, in the VIC-3 group. On the contrary, the expression profile of these genes was almost undetectable in the other four groups. Particularly concerning the expression profiles in the Fraoula-related groups of the *otaR1* gene, which encodes a bZIP TF playing a pivotal role in the OTA biosynthetic pathway by regulating the other OTA cluster genes [13], a slightly higher expression was revealed in FRA-2 compared to the FRA-1 and FRA-3 groups. Furthermore, in terms of the *LaeA* gene, which is a global nuclear methyltransferase regulating the biosynthesis of secondary metabolites required for OTA biosynthesis, and in terms of the *GOX* gene that regulates the GLA accumulation and acidification of the host tissue during *A. carbonarius* growth [79], both were mainly expressed in the VIC-3 group. Previously, *A. carbonarius* ∆*laeA* and ∆*gox* mutants produced less GLA and were unable to effectively acidify the colonized tissue, which resulted in attenuated virulence and pathogenicity in the infected grape berry [79]. Notably, it is worth mentioning that the *GOX* was among the highest detected genes of *A. carbonarius* in all six comparison groups, mainly in the three Victoria-related groups. Although OTA accumulation does not necessary contribute to enhancing *A. carbonarius* pathogenicity [79], our data allow us to speculate that the increased transcriptional motivation of the OTA-biosynthesis gene cluster in the Victoria variety is positively related with the higher fungal growth and conidiation in this variety. In line with this, a higher OTA accumulation was observed in the Victoria variety than in the Fraoula variety upon the seven-day inoculation period.

## 4. Materials and Methods

### 4.1. Fungal Isolate and Grape Inoculation

The *A. carbonarius* strain Ac29 that belongs to the fungal culture collection of the Laboratory of Food Microbiology and Biotechnology (LFMB) of the Agricultural University of Athens (AUA) was used for grape inoculation. This fungus was previously isolated from grape bunches sampled in Greek vineyards and was molecularly characterized [82]. Two table grape varieties (Victoria and Fraoula, white and red variety, respectively) were sampled during the harvest ripening stage in 2023 from commercial vineyards located close to each other in the Attica province (central Greece). They were cultivated under the same standard cultural practices.

Homogenous in size and color and with no mechanical or biological damage visible on their skin, berries were randomly selected from five bunches for each variety. The artificial detached-berry pathogenicity assay was conducted with the methodology as previously described [83]. Briefly, the berries were initially surface-disinfected using 0.05% sodium hypochlorite for 10 min, rinsed three times with sterile distilled water, and left to air-dry in a sterilized bench. The inoculum was prepared by growing the Ac29 strain in Petri dishes filled with potato dextrose agar (PDA) for 7 days at 25 °C. At the end of incubation, Petri dishes were washed with 10 mL of sterile distilled water, and conidia were counted using a Burker chamber. The fungal inoculation was performed by immersing the berries of both varieties for 5 min in a spore suspension of *A. carbonarius* adjusted at a concentration of 10^6^ spores mL^−1^ with the addition of 0.05% tween 20 (AC treatments; VIC-AC and FRA-AC treatments for the Victoria and Fraoula varieties, respectively). Mock-inoculated control berries were also surface-disinfected as above, and were immersed in aqueous solution with addition of 0.05% tween 20 (CT treatments; VIC-CT and FRA-CT treatments for the Victoria and Fraoula varieties, respectively). Then, all berries were placed inside sterile plastic boxes bearing a perforated metal base on which the berries stand and, underneath, 2 pieces of damp paper were placed, fully wet (15 mL of sterile water). Thus, the plastic boxes are to be used as humid chambers. The boxes were incubated at 25 °C with 12 h light and 12 h darkness. For all four treatments, samples were taken at three time points (1, 2, and 3 dai) with 3 replicates per treatment consisting of a pool of 10 berries for each time point and variety. The samples were snap-stored in deep freeze (−80 °C) for later use in the transcriptomic analyses.

### 4.2. Fungal and OTA Quantification

For both fungal and OTA quantification, the same methodology as above was followed in both AC treatments (VIC-AC and FRA-AC). Particularly, berries upon *A. carbonarius* inoculation were incubated at 25 °C with 12 h light and 12 h darkness for 7 days. At the end of this incubation period, fungal and OTA quantification were performed in grape berries after their homogenization with the Ultra Turrax mixer (T25 basic IKA-Werke, Staufen, Germany) using three replicates, each consisting of 10 pool berries. The juice obtained was used to quantify fungal conidia per mL using a haemocytometer. In addition, the grape juice for the OTA quantification was extracted using an equal volume of 70% methanol as indicated in the commercial ELISA kit Agra-Quant Ochratoxin A (RomerLabs, Getzersdorf, Austria). The assays were conducted based on the procedure described in the Agra-Quant Assay kit manual. Using a 450 nm absorbance filter, the amount of absorption in microwells was determined using a microwell reader (BioTek 800 TS, Winooski, VT, USA). The standard curve that was created in accordance with the kit’s instructions was used to report the results in μg/L (ppb). The statistical analysis was conducted by performing one-way ANOVA and the significance across treatments was detected by Tukey’s multiple-comparison post hoc test (*p* < 0.05).

### 4.3. RNA-Seq Analysis and Bioinformatics

In order to decipher the modes of action of transcriptomic profiles in the two grape varieties after *A. carbonarius* challenge, a dual RNA-seq analysis was conducted upon 36 berry samples. Thus, samples consisted of berries inoculated with the pathogen (VIC-AC and FRA-AC treatments) or from untreated, mock-inoculated berries (VIC-CT and FRA-CT treatments), employing three biological replicates for each treatment across three time points upon inoculation, 1, 2, and 3 days after inoculation (dai). Thus, total RNA from the 36 samples was extracted using the Plant/Fungi Total RNA Purification Kit (Norgen Biotek Corp., Thorold, Ontario, Canada). Sequencing libraries were constructed by the PT042 NGS RNA Library Prep Set (Novogene Ltd., Cambridge, UK). RNA-seq was performed using the Illumina (San Diego, CA, USA) Novaseq 6000 sequencing platform, producing 2 × 150 bp paired-end (PE) reads. The Pinot Noir 12X genomic assembly (accession GCF_000003745.3) and its gene models were employed to map the clean PE reads with the HISAT2 software (v2.0.5) with default settings [84]. The FPKM method was used to mount the levels of gene expression, and the DESeq2 R package (1.20.0) was employed to identify differentially expressed genes (DEGs) [85] based on an absolute value of log2foldchange ≥ 1 with an adjusted *p*-value ≤ 0.05. Using a model based on the negative binomial distribution, this method offers statistical routines for identifying differential expression in digital gene expression data. The *p*-values that resulted were then adjusted for multiple testing using Benjamini and Hochberg’s false discovery rate (FDR) control procedure. Then, gene ontology (GO) terms were subjected to an enrichment analysis using the clusterProfiler R package (3.8.1), while GO terms with a *p*-value < 0.05 were classified as significantly enriched [86]. This package uses combined analysis and visualization modules to automate the process of classifying GO terms and enriching gene clusters. DEGs were also classified according to KEGG-orthology-enriched terms, as was previously reported [47]. Reads mapped to the *A. carbonarius* transcriptome were identified using the fungal-inoculated berries samples (VIC-AC and FRA-AC treatments). Thus, the unmapped reads against the grape genome were subsequently mapped to the *A. carbonarius* (strain ITEM 5010) GCA_001990825.1 reference genomic assembly with the HISAT2 software (v2.0.5) with default settings [84]. The normalized transcript counts were estimated using the DESeq2 R package (1.20.0) while the reads were assigned at the transcript level using the htseq-count software (v2.0.2) with default settings [87].

### 4.4. Gene Expression Validation

The relative gene expression of six grape DEGs was confirmed by quantitative real-time PCR (RT-qPCR) analysis, to validate the RNA-seq data. A reference gene (LOC100232866) encoding for actin was employed to normalize the expression profiles of PCR reactions, which were run in triplicate. To track the relative quantitative expression ratios of the inoculated samples compared to the corresponding controls, the 2^−△△CT^ method was employed [88].

## 5. Conclusions

Transcriptional profiles, OTA contamination, and fungal growth were investigated in grape berries of two varieties upon inoculation with *A. carbonarius*. The results highlighted a dynamic and time-dependent transcriptional reprogramming which was also variety-dependent during the infection period. Thus, DEGs related to the activation of defense responses, the metabolic shunt for the biosynthesis of secondary metabolites and phytoalexins, the induction of TFs and genes involved in plant–pathogen interaction, and immune signaling transduction were revealed in an early time point in the red variety, whereas, in the white variety, the transcriptional responses were generally delayed. Even though these were distinct expression profiles, both varieties revealed, to some extent, common expression dynamics for specific DEGs families, such as those encoding for laccases and stilbene synthases. A critical modulator role in the recruiting defense machinery of grape berries against *A. carbonarius* seems to be inferred by the activation of various JA-biosynthetic DEGs. Furthermore, the majority of DEGs associated with an increasing sensitivity to the pathogen were mainly activated in the white variety. This is consistent with the significantly higher OTA accumulation and fungal development in terms of the conidia quantification observed in the white variety. In line with this, the expression profiles of specific *A. carbonarius* genes related to pathogenesis, fungal sporulation, and conidiation highlight the higher susceptibility of the white variety. It is also worth mentioning that *A. carbonarius* transcriptional patterns which are directly associated with the regulation of the pathogen OTA-biosynthesis gene cluster were more highly induced in the white variety. This study improves our understanding of grape variety resistance to *A. carbonarius* at transcriptional levels.

## Data Availability

The datasets generated during the current study are available in the NCBI SRA database below: https://www.ncbi.nlm.nih.gov/, PRJNA1043429.

## Supplementary Materials

The following supporting information can be downloaded at https://www.mdpi.com/article/10.3390/plants13152043/s1. Figure S1: Volcano plots showing the distribution of differentially expressed genes (DEGs) across the six comparison groups (VIC-1, VIC-2, VIC-3, FRA-1, FRA-2, and FRA-3) at three time points (dai) upon inoculation with *A. carbonarius*; Figure S2: Enrichment scatter plots based on DEG gene ontology (GO)-based functional categorizations across the six comparison groups (VIC-1, VIC-2, VIC-3, FRA-1, FRA-2, and FRA-3). The counts of the DEGs being annotated in the corresponding GO terms are shown, whereas the significant size (adjusted *p*-value, padj) of the enrichment is indicated by a color; Figure S3: Correlation of RT-qPCR and RNA-seq log2foldchange expression values of six randomly selected genes in the VIC-2 comparison group after *A. carbonarius* inoculation (2 dai) on grape berries; Table S1: Summary of grape berries’ RNA-seq data; Table S2: Differentially expressed gene (DEG) profiles of grape berries across the six comparison groups (VIC-1, VIC-2, VIC-3, FRA-1, FRA-2, and FRA-3) after *A. carbonarius* inoculation; Table S3: Differentially expressed gene (DEG) profiles of selected key genes involved in cell-wall modification and degradation across the six comparison groups (VIC-1, VIC-2, VIC-3, FRA-1, FRA-2, and FRA-3) after *A. carbonarius* inoculation; Table S4: Differentially expressed gene (DEG) profiles of selected key genes involved in pathogen perception and signaling transduction across the six comparison groups (VIC-1, VIC-2, VIC-3, FRA-1, FRA-2, and FRA-3) after *A. carbonarius* inoculation; Table S5: Differentially expressed gene (DEG) profiles of selected key genes encoding pathogenesis and defense-related proteins across the six comparison groups (VIC-1, VIC-2, VIC-3, FRA-1, FRA-2, and FRA-3) after *A. carbonarius* inoculation; Table S6: Differentially expressed gene (DEG) profiles of selected key genes encoding transcriptional factors (TFs) across the six comparison groups (VIC-1, VIC-2, VIC-3, FRA-1, FRA-2, and FRA-3) after *A. carbonarius* inoculation; Table S7: Differentially expressed gene (DEG) profiles of selected key genes involved in secondary and primary metabolism across the six comparison groups (VIC-1, VIC-2, VIC-3, FRA-1, FRA-2, and FRA-3) after *A. carbonarius* inoculation; Table S8: Differentially expressed gene (DEG) profiles of selected key genes encoding nutrient and ion transporters across the six comparison groups (VIC-1, VIC-2, VIC-3, FRA-1, FRA-2, and FRA-3) after *A. carbonarius* inoculation; Table S9: *A. carbonarius*-expressed genes and average counts of normalized reads across the six comparison groups (VIC-1, VIC-2, VIC-3, FRA-1, FRA-2, and FRA-3) after the pathogen inoculation on grape berries; Table S10: Specific primers used in RT-qPCR for the validation of RNA-seq data.
