# Supplementary material for: Unlocking the Transcriptional Reprogramming Repertoire between Variety-Dependent Responses of Grapevine Berries to Infection by Aspergillus carbonarius
Source: Plants (Basel). 2024 Jul 25;13(15):2043. doi: 10.3390/plants13152043 (PMC11314482; doi:10.3390/plants13152043)
Supplement: Supplementary file 1 [file plants-13-02043-s001.zip › Kavroumatzi_Supplementary_Figures_Plants.pdf]

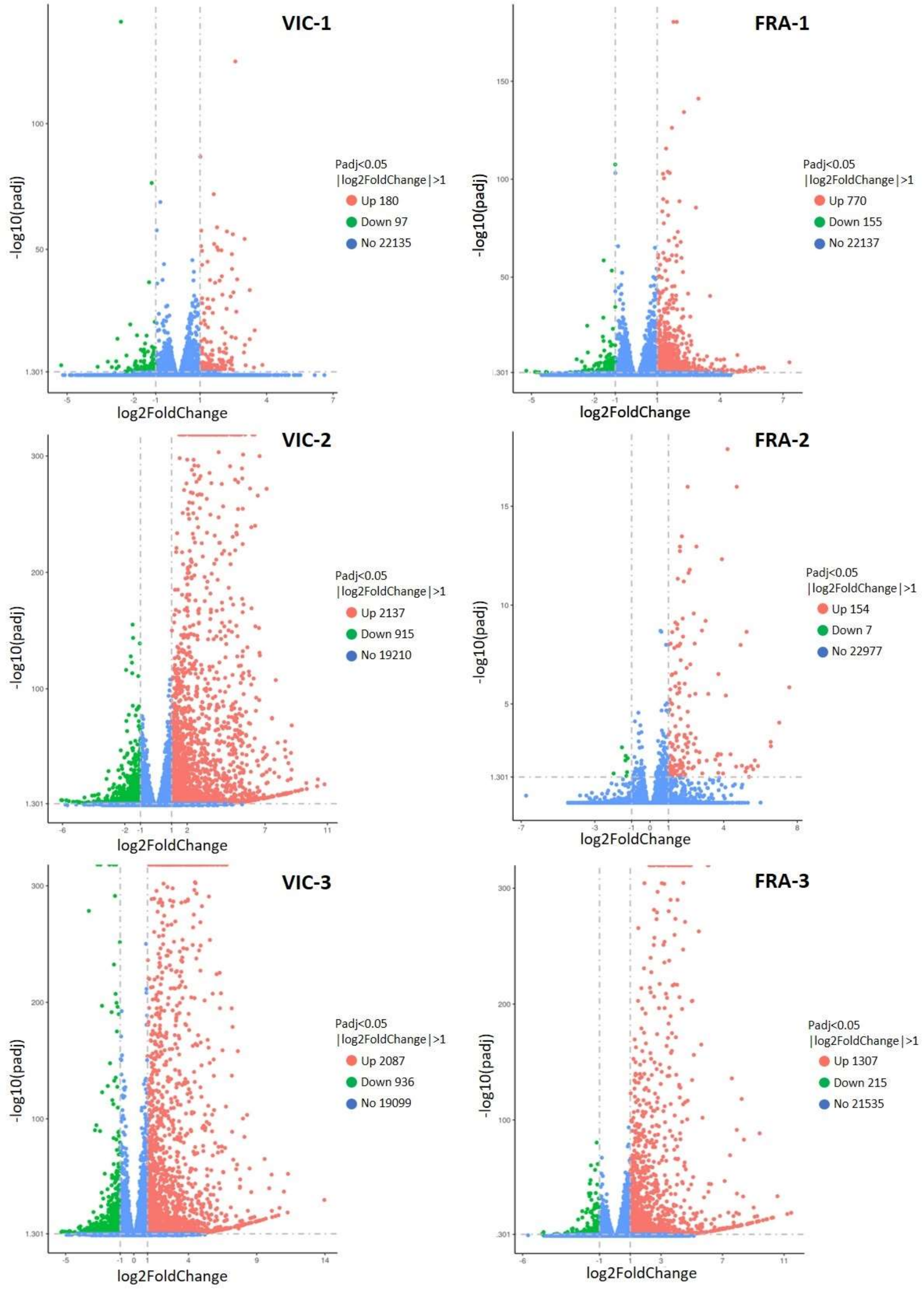


Figure S1: Volcano plots showing the distribution of differentially expressed genes (DEGs) across the six comparison groups (VIC-1, VIC-2, VIC-3, FRA-1, FRA-2, FRA-3) at three time points (dai) upon inoculation with *A. carbonarius*.

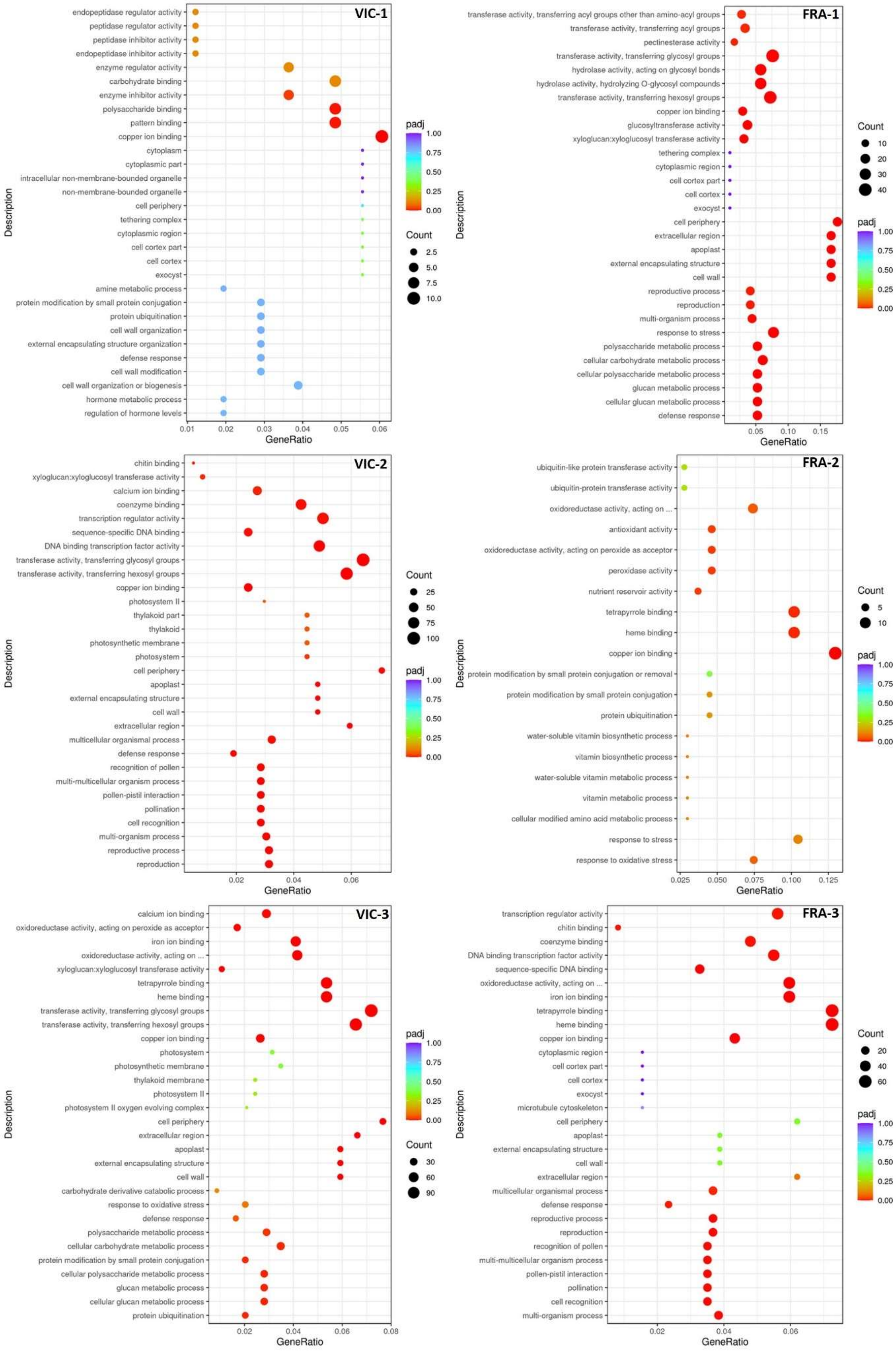


Figure S2: Enrichment scatter plots based on DEGs gene ontology (GO)-based functional categorizations across the six comparison groups (VIC-1, VIC-2, VIC-3, FRA-1, FRA-2, FRA-3). The counts of the DEGs being annotated in the corresponding GO terms are shown, whereas the significant size (adjusted P-value, padj) of the enrichment is indicated by a color.

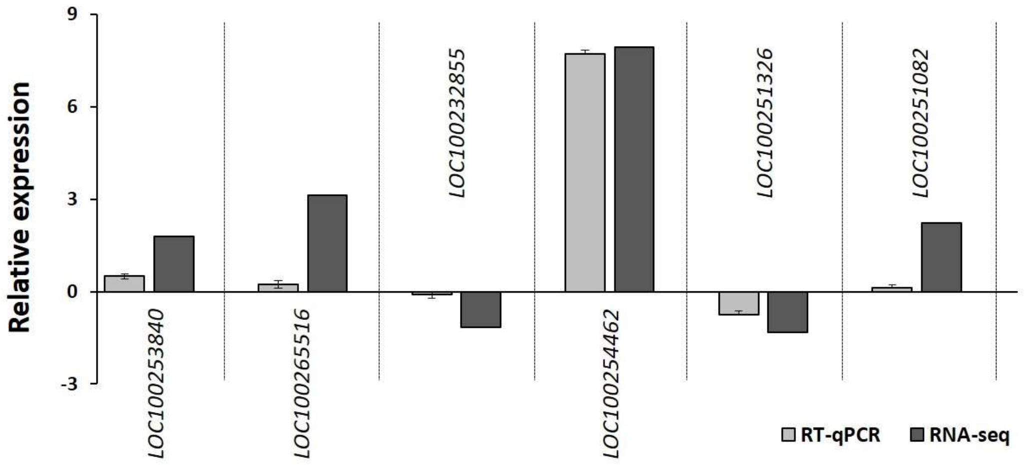


Figure S3: Correlation of RT-qPCR and RNA-seq log2foldchange expression values of six randomly selected genes in the VIC-2 comparison group after *A. carbonarius* inoculation (2 dai) on grape berries.
