# Supplementary figures and images for: Unlocking the Transcriptional Reprogramming Repertoire between Variety-Dependent Responses of Grapevine Berries to Infection by Aspergillus carbonarius
Source: Plants (Basel). 2024 Jul 25;13(15):2043. doi: 10.3390/plants13152043 (PMC11314482; doi:10.3390/plants13152043)

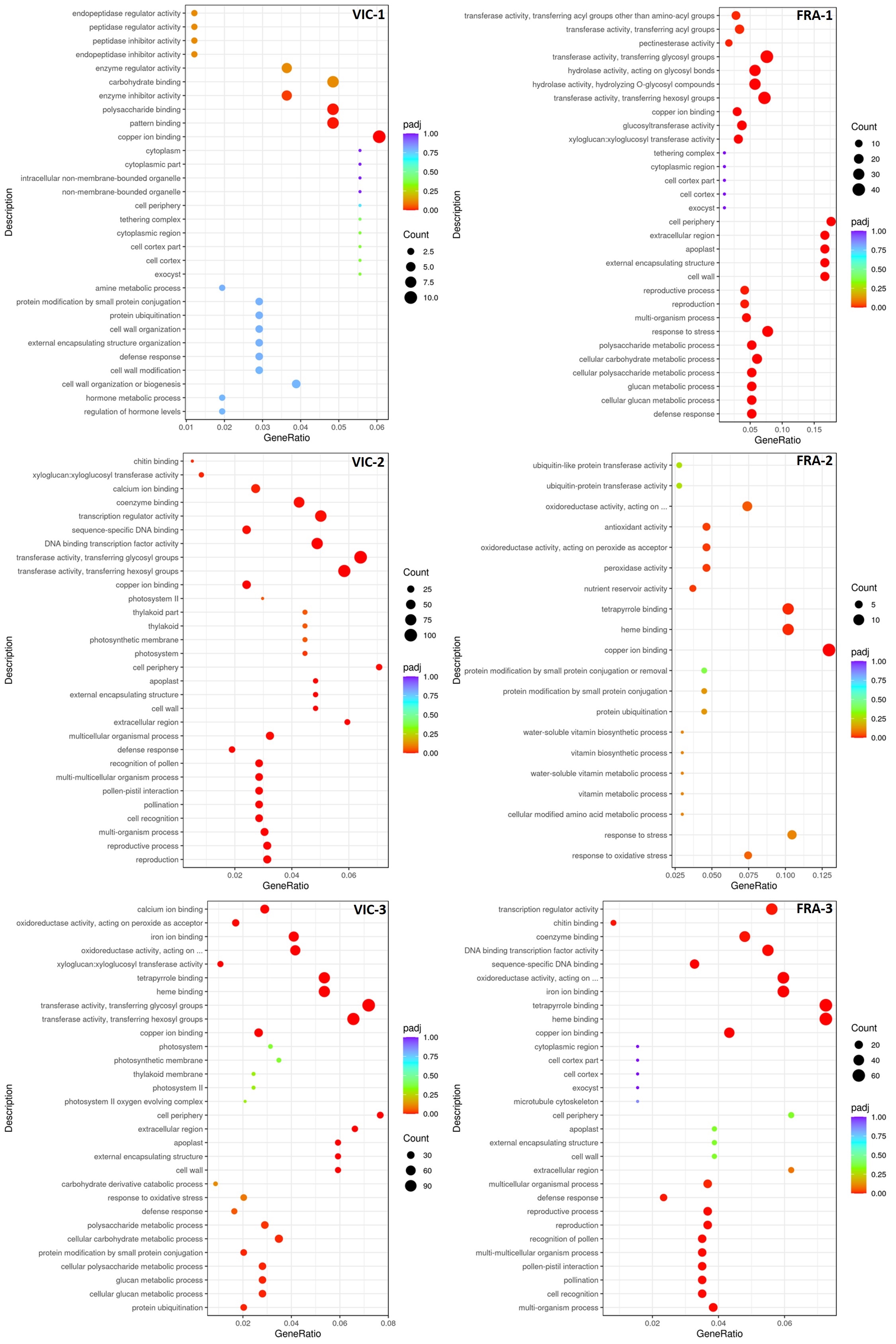

Supplement: Supplementary file 1 [file plants-13-02043-s001.zip › Figure S2.jpg]

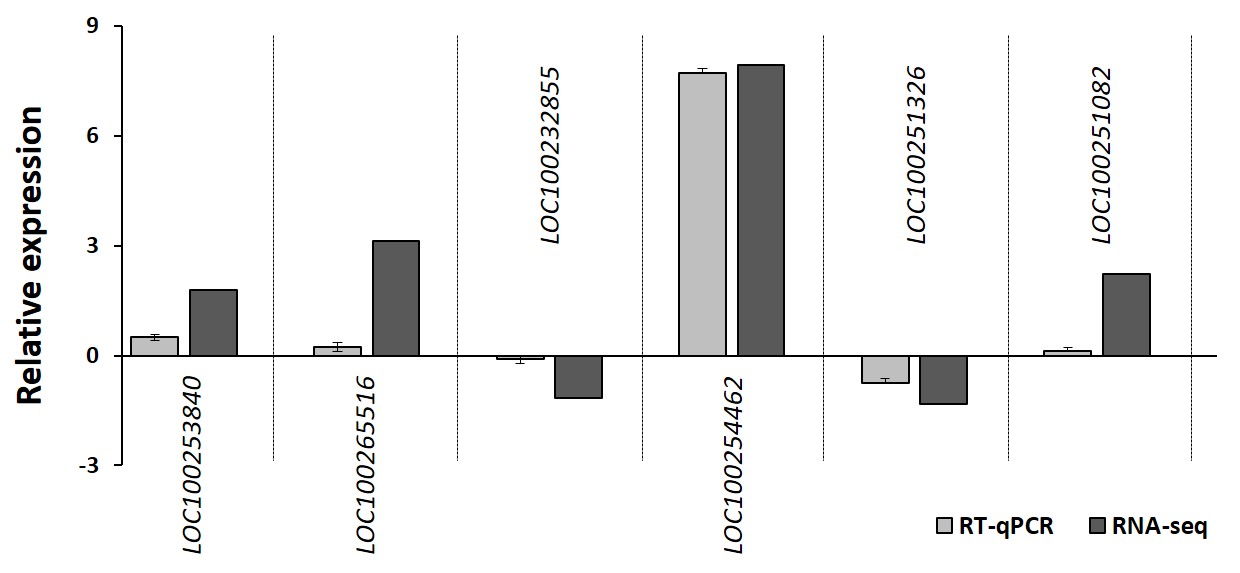

Supplement: Supplementary file 1 [file plants-13-02043-s001.zip › Figure S3.jpg]

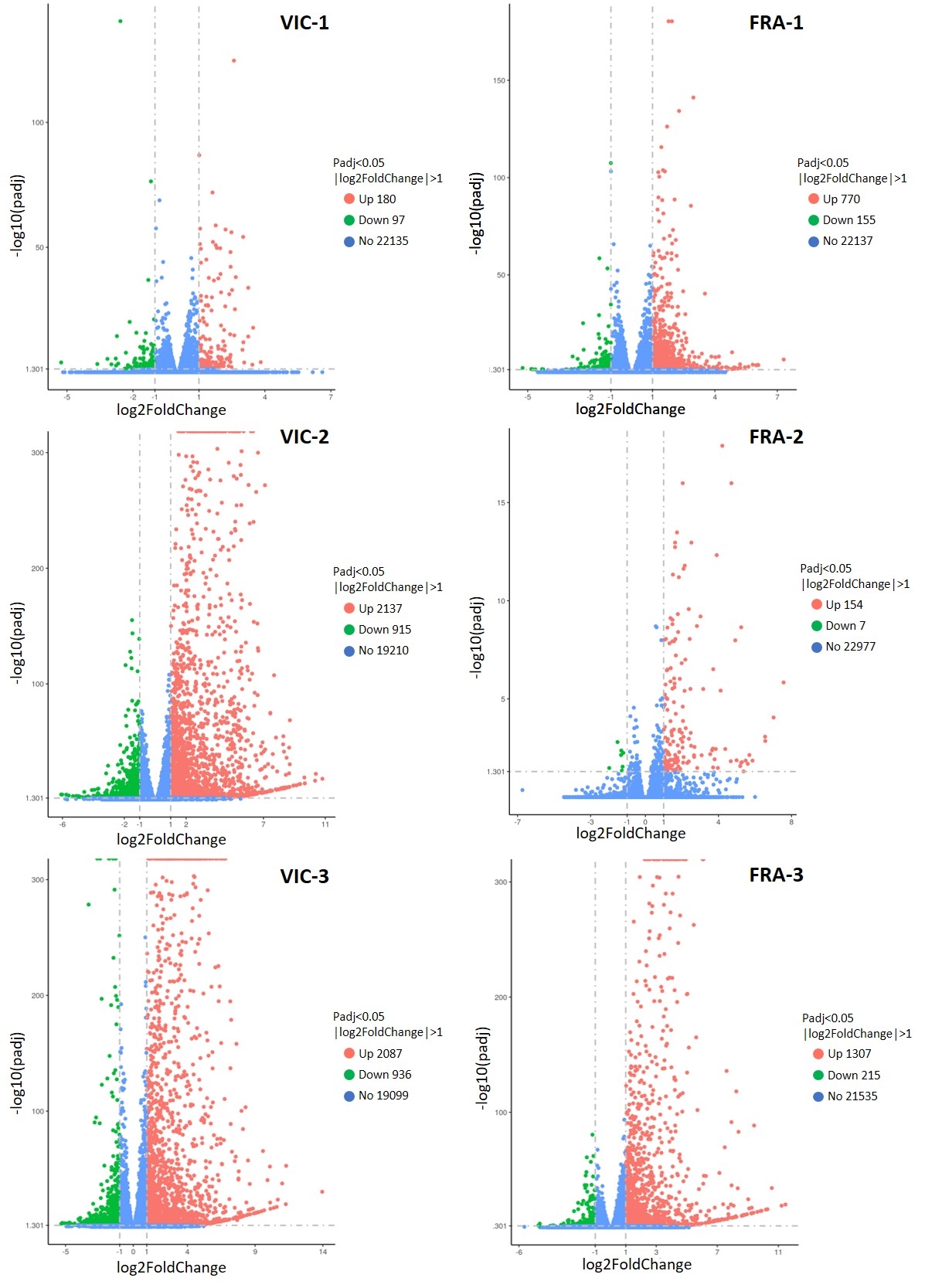

Supplement: Supplementary file 1 [file plants-13-02043-s001.zip › Figure S1.jpg]
